# Supplementary material for: Longitudinal Analysis of Humoral and Cellular Immune Response up to 6 Months after SARS-CoV-2 BA.5/BF.7/XBB Breakthrough Infection and BA.5/BF.7-XBB Reinfection
Source: Vaccines (Basel). 2024 Apr 26;12(5):464. doi: 10.3390/vaccines12050464 (PMC11125724; doi:10.3390/vaccines12050464)
Supplement: Supplementary file 1 [file vaccines-12-00464-s001.zip › vaccines-2946977-supplementary.pdf]

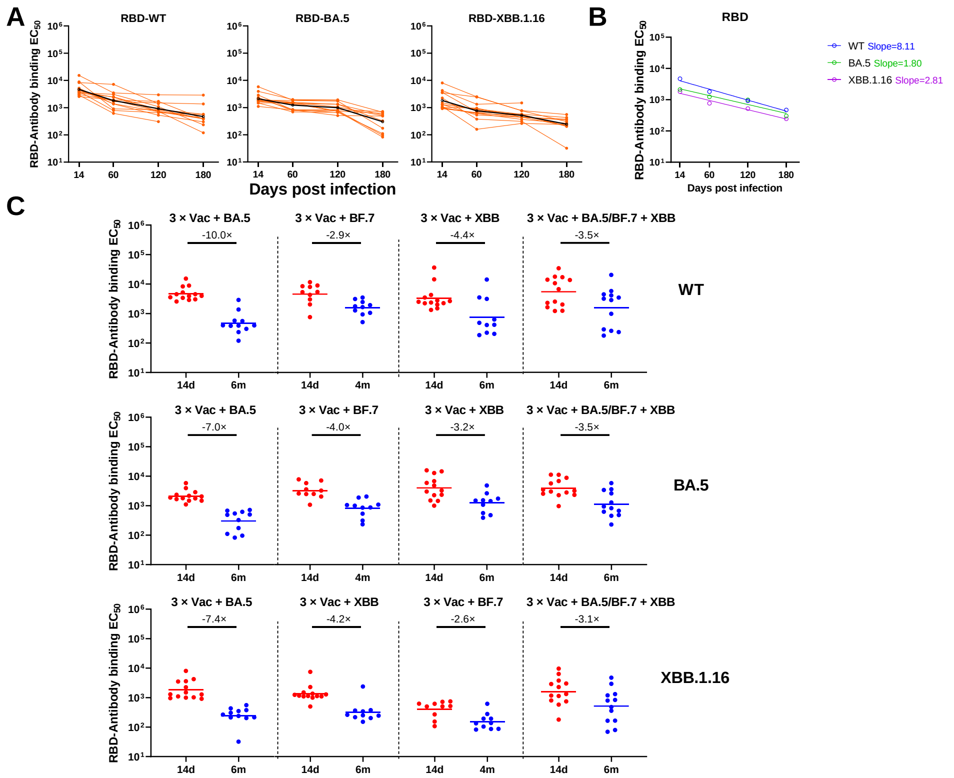

Supplementary Figure S1. (A) Serum anti-WT/BA.5/XBB.1.16 RBD IgG levels over time for individuals with BA.5 breakthrough infection. (B) Decline rate of binding GMTs by sera collected from individuals with BA.5 breakthrough infection against WT/BA.5/XBB.1.16 RBD over time. (C) Parallel comparison of anti-WT/BA.5/XBB.1.16 RBD IgG levels at Day 14 and 4 or 6 months after BA.5/BF.7/XBB breakthrough infection or BA.5/BF.7-XBB reinfection.

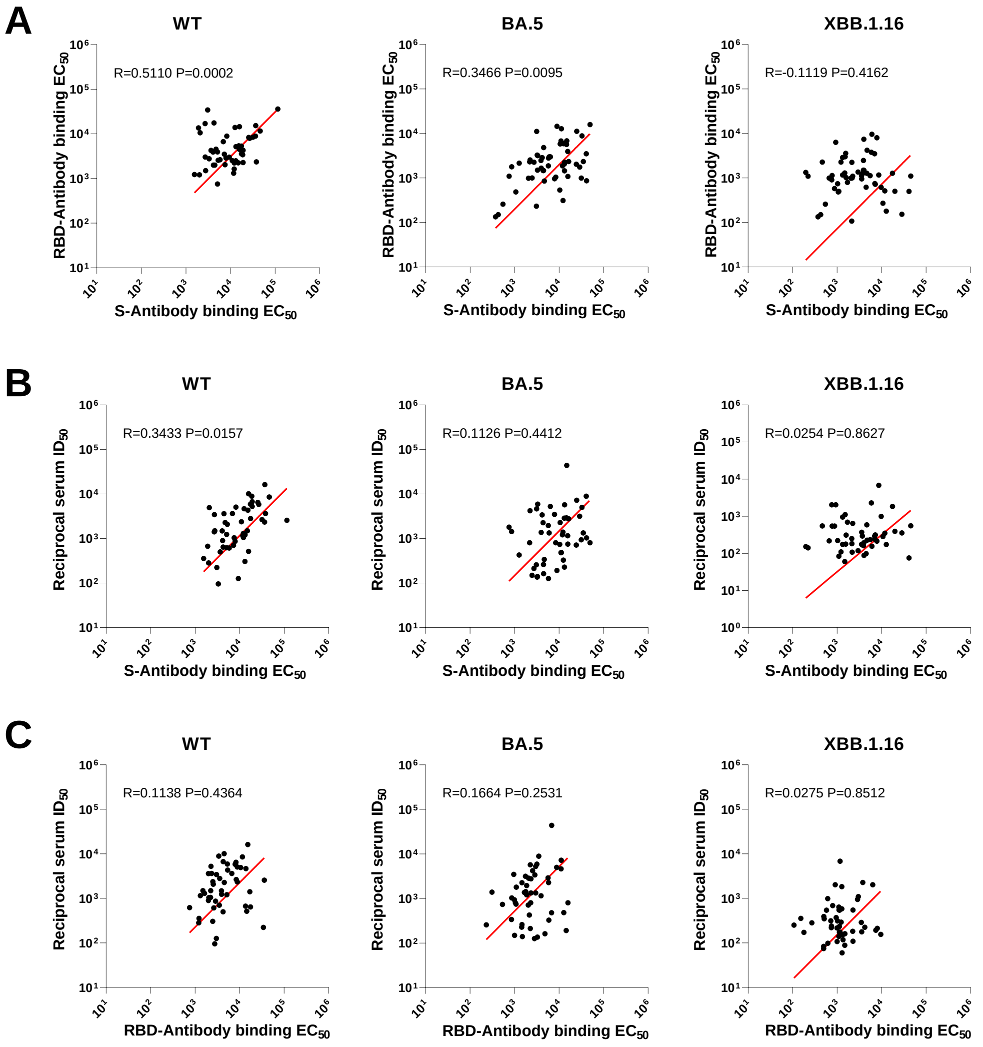

Supplementary Figure S2. (A) The correlation between anti-WT/BA.5/XBB.1.16 Spike and RBD IgG EC_{50} data from BA.5/BF.7/XBB breakthrough infection and BA.5/BF.7-XBB reinfection groups at Day 14. The correlation between anti-WT/BA.5/XBB.1.16 Spike (B) and RBD (C) IgG and neutralization ID_{50} combining data from BA.5/BF.7/XBB breakthrough infection and BA.5/BF.7-XBB reinfection groups at Day 14. The R value represents the correlation coefficient. Statistics were calculated using Spearman's rank correlation.

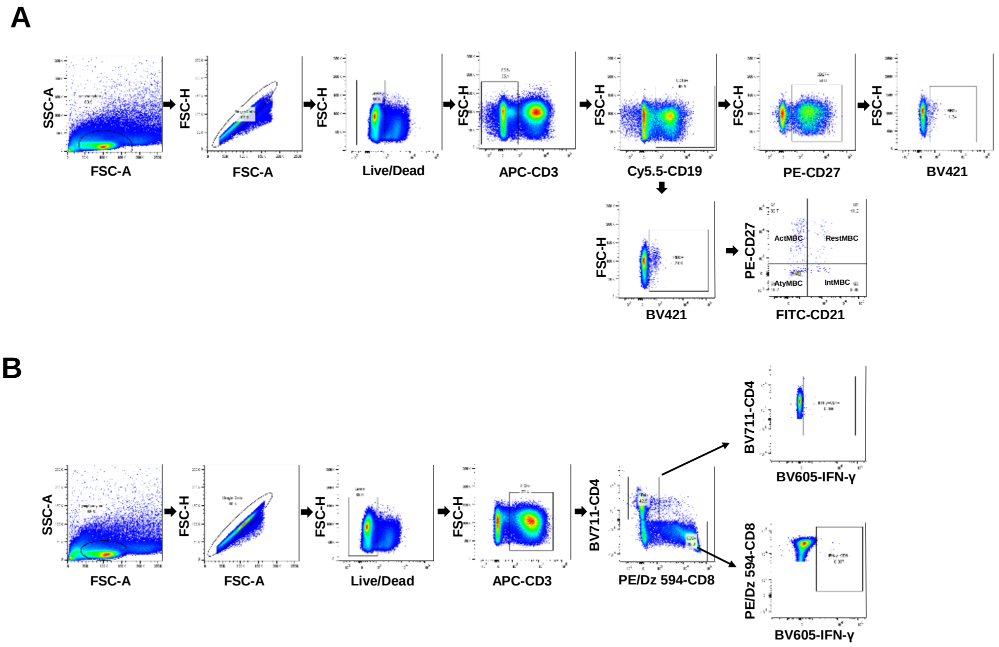

Supplementary Figure S3. Gating strategies and control experiments for Cellular immunity investigations. The full strategies for gating RBD-specific memory B cells (A) and S-specific T cells (B) in flow cytometry. actMBCs, activate memory B cells; atyMBCs, atypical memory B cells; intMBCs, intermediate memory B cells; rMBCs, resting memory B cells.

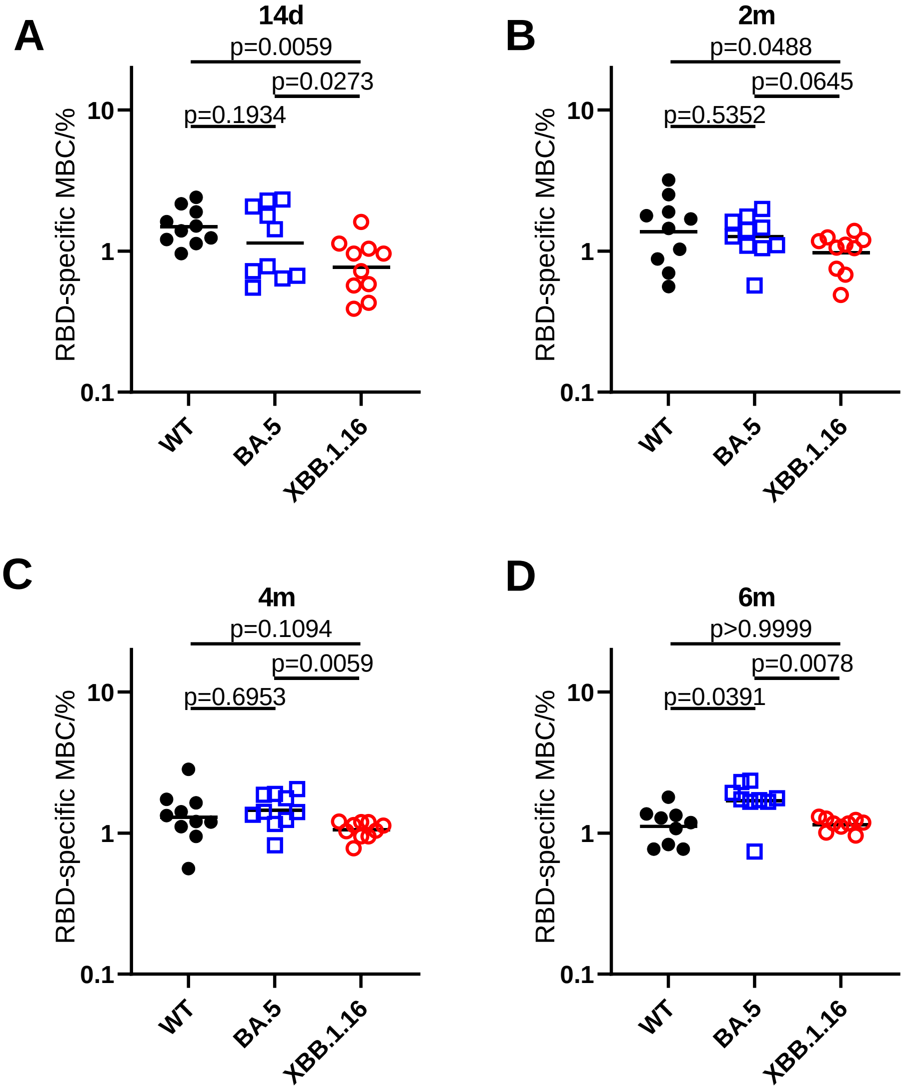

Supplementary Figure S4. Comparison of the frequencies of WT/BA.5/XBB.1.16 RBD-specific MBCs from individuals with BA.5 breakthrough infections at 14d (A), 2m (B), 4m (C), 6m (D).

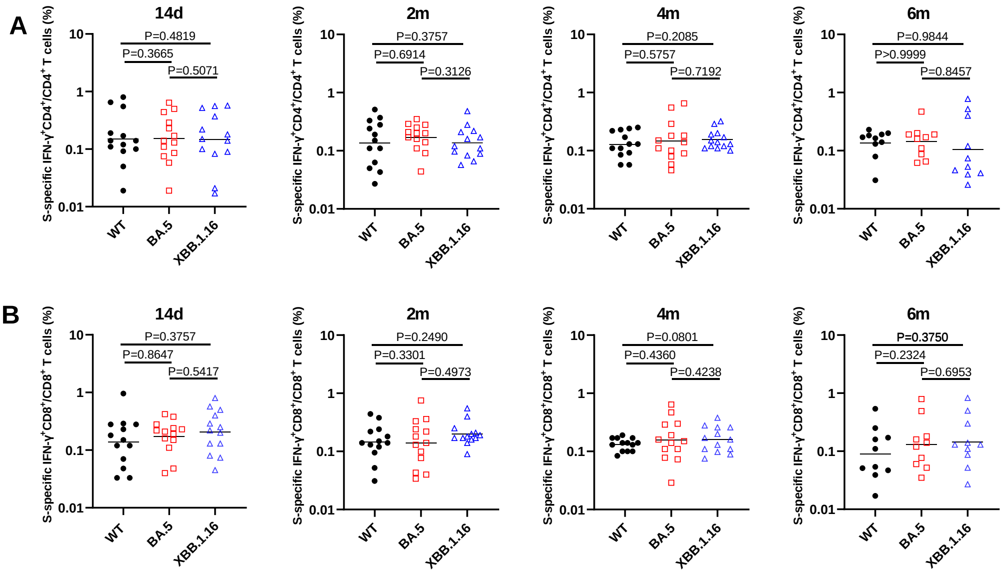

Supplementary Figure S5. Comparison of the frequencies of WT/BA.5/XBB.1.16 S-specific IFN-γ$^+$CD4$^+$T cells (A) or IFN-γ$^+$CD8$^+$T cells (B) from individuals with BA.5 breakthrough infections at 14d, 2m, 4m, 6m.
